# A H_2_O_2_‐Supplied Supramolecular Material for Post‐irradiated Infected Wound Treatment

**DOI:** 10.1002/advs.202206851

**Published:** 2023-01-29

**Authors:** Peidong Du, Yanzhe Shen, Baoli Zhang, Shan Li, Minzheng Gao, Ting Wang, Xiaokang Ding, Bingran Yu, Zhen‐Gang Wang, Fu‐Jian Xu

**Affiliations:** ^1^ State Key Laboratory of Organic‐Inorganic Composites Key Laboratory of Biomedical Materials of Natural Macromolecules (Beijing University of Chemical Technology, Ministry of Education) Beijing Laboratory of Biomedical Materials Beijing University of Chemical Technology Beijing 100029 P. R. China; ^2^ CAS Key Laboratory for Biomedical Effects of Nanomaterials and Nanosafety National Center for Nanoscience and Technology Beijing 100190 P. R. China

**Keywords:** antibacterial therapy, G‐quartet, post‐irradiation, riboflavin, self‐assembly

## Abstract

Photodynamic therapy (PDT) is a light triggered therapy by producing reactive oxygen species (ROS), but traditional PDT may suffer from the real‐time illumination that reduces the compliance of treatment and cause phototoxicity. A supramolecular photoactive G‐quartet based material is reported, which is self‐assembled from guanosine (G) and 4‐formylphenylboronic acid/1,8‐diaminooctane, with incorporation of riboflavin as a photocatalyst to the G4 nanowire, for post‐irradiation photodynamic antibacterial therapy. The G4‐materials, which exhibit hydrogel‐like properties, provide a scaffold for loading riboflavin, and the reductant guanosine for the riboflavin for phototriggered production of the therapeutic H_2_O_2_. The photocatalytic activity shows great tolerance against room temperature storage and heating/cooling treatments. The riboflavin‐loaded G4 hydrogels, after photo‐irradiation, are capable of killing gram‐positive bacteria (e.g., *Staphylococcus aureus*), gram‐negative bacteria (e.g., *Escherichia coli*), and multidrug resistant bacteria (methicillin‐resistant *Staphylococcus aureus*) with sterilization ratio over 99.999%. The post‐irradiated hydrogels also exhibit great antibacterial activity in the infected wound of the rats, revealing the potential of this novel concept in the light therapy.

## Graphical abstract

A post‐irradiation photodynamic therapy is presented that has no requirement for confinement of the patients to the illumination source. Supramolecular riboflavin‐loaded G4‐hydrogels are triggered by blue light to catalytically generate H_2_O_2_, which is then subjected to the infected wound for killing bacteria.

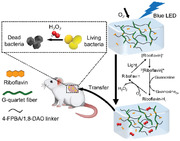

## 1 Introduction

Photodynamic therapy (PDT),^[^ ^1^, ^2^, ^3^, ^4^, ^5^, ^6^ ^]^ as a minimally invasive and cost‐effective therapeutic modality, has gained considerable attention in the treatment of a range of cancers,^[^ ^7^, ^8^, ^9^ ^]^ wound infection,^[^ ^10^ ^]^ and other nonmalignant diseases.^[^ ^11^ ^]^ PDT uses photosensitizers that react with the molecular oxygen,^[^ ^12^, ^13^ ^]^ upon activated with specific‐wavelength light, to generate reactive oxygen species (ROS) in the target tissue, to elicit the cell death. However, the traditional PDT suffered from short half‐life of ROS (e.g., ^1^O_2_, ≈10^−5^ s; ∙OH, ≈10^−9^ s; superoxide anion, ≈5 s),^[^ ^14^, ^15^ ^]^ which have extremely high reactivity but decay and degrade fast as a result of chemical and physical quenching. The short lifespan of the ROS leads to very short diffusion path in vivo (e.g., ^1^O_2_, less than 270 nm).^[^ ^16^ ^]^ Moreover, the patients have to be confined to the irradiation source for in‐site ROS generation, which reduces the patients‘ comfort, and causes potential phototoxicity, and limited the clinical application of PDT. To prolong the ROS duration, molecular hosts have been used to capture the ROS species,^[^ ^17^, ^18^ ^]^ and to release them to the target, which has been employed for overcoming the photoinduced hypoxia. This approach can be applied to avoid real‐time illumination, but may suffer from incomplete ROS capturing and releasing. On the other hand, as an antiseptic ROS reagent, H_2_O_2_ has been widely used in biomedical field, particularly in wound cleaning by killing pathogens through oxidation burst^[^ ^19^ ^]^ and local oxygen radical production.^[^ ^20^, ^21^ ^]^ On‐demand generation of H_2_O_2_ at the infectious wound site, which can be achieved by photo‐irradiation of the sensitizers, would minimize its overuse and the adverse effect on the normal cells, but has been rarely reported. The mild reactivity of the as‐generated H_2_O_2_ allows it to be stored in the therapeutic systems during the photo‐irradiation, and then subjected to patients, so that the postirradiation PDT therapy may be achieved.

**Figure 1:**
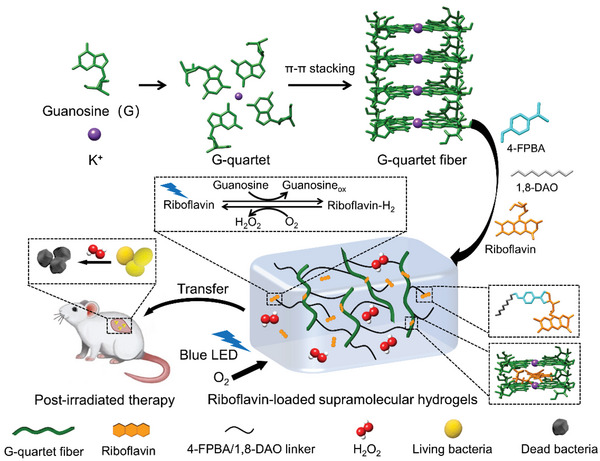
Schematic formation of the riboflavin‐loaded supramolecular hydrogels for post‐irradiation antibacterial therapy.

In this work, we combined photocatalytic flavin‐based molecular catalyst with the guanosine‐derived supramolecular materials for the antibacterial activity (**Figure** **1**). In the presence of K^+^, the guanosine forms the G‐quartet motifs (G4) via Hoogsteen base pairing, which stack into G4 wires.^[^ ^22^, ^23^ ^]^ With assistance of the 4‐formylphenylboronic acid (4‐FPBA) and 1,8‐diaminooctane, G4 are crosslinked to form gel‐like materials.^[^ ^24^, ^25^ ^]^ The flavin derivatives (e.g., riboflavin) are loaded to the hydrogels via chemical conjugation and stacking interactions, to form the photocatalytic supramolecular materials. The photosensitive flavin moiety can be excited upon light irradiation to catalyze the oxidation of the reductant (e.g., guanosine), followed by O_2_ reduction to H_2_O_2_.^[^ ^26^, ^27^ ^]^

Herein, the guanosine‐derived supramolecular materials act as the dressing materials for the wound, and provides the guanosine as the chemical fuels for generating the therapeutic H_2_O_2_. We have found the riboflavin‐loaded supramolecular materials were capable of killing gram‐positive bacteria (e.g., *Staphylococcus aureus*), gram‐negative bacteria (e.g., *Escherichia coli*) and multidrug resistant bacteria (e.g., methicillin‐resistant *S. aureus*, MRSA), after irradiation with blue light. The post‐irradiation therapy may also avoid the photocytotoxity that arise from the damage of high‐energy light to the normal cells.

## 2 Result and Discussion

**Figure 2:**
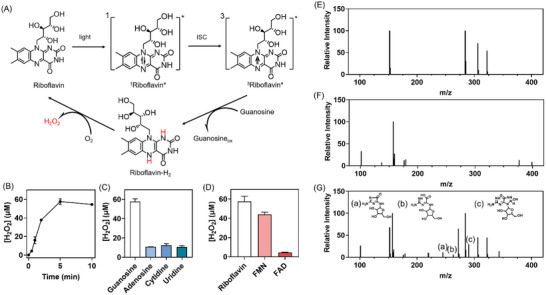
A) Catalytic cycle of riboflavin mediated photo‐oxidation and H_2_O_2_ production. B) The amount of H_2_O_2_ generated by riboflavin mediated photo‐oxidation of guanosine with different irradiation time. (*N* = 3, data are shown as the mean ± standard deviation). [riboflavin]: 10 × 10^−6^ m, [guanosine]: 200 × 10^−6^ m, [HRP]: 10 × 10^−9^ m, [TMB]: 0.3 × 10^−3^ m, [PBS]: 20 × 10^−3^ m, pH 7.4. C) The amount of H_2_O_2_ generated by photo‐oxidation of different nucleosides (*N* = 3, data are shown as the mean ± standard deviation). [riboflavin]: 10 × 10^−6^ m, [nucleoside]: 200 × 10^−6^ m, [HRP]: 10 × 10^−9^ m, [TMB]: 0.3 × 10^−3^ m, [PBS]: 20 × 10^−3^ m, pH 7.4. D) The amount of H_2_O_2_ generated by different photosensitizers mediated photo‐oxidation of guanosine (*N* = 3, data are shown as the mean ± standard deviation). [photosensitizer]: 10 × 10^−6^ m, [guanosine]: 200 × 10^−6^ m, [HRP]: 10 × 10^−9^ m, [TMB]: 0.3 × 10^−3^ m, [PBS]: 20 × 10^−3^ m, pH 7.4. E–G) ESI‐MS spectrum: (E) guanosine, (F) riboflavin, and (G) guanosine/riboflavin after blue light irradiation. [riboflavin]: 50 × 10^−6^ m, [guanosine]: 1 × 10^−3^ m.

As Figure S1 (Supporting Information) shows, riboflavin has a strong absorption peak around 460 nm. Upon irradiation by blue light (460 nm), riboflavin is excited to singlet state and rapidly converts to triplet with high oxidation potential through intersystem crossing (ISC).^[^ ^26^ ^]^ The triplet state as the active species is then reduced by the nucleoside to dihydro‐riboflavin (Riboflavin‐H_2_),^[^ ^26^, ^28^ ^]^ which is oxidized by O_2_ to form the resting state. Meanwhile, O_2_ is reduced to generate H_2_O_2_ (**Figure** **2**A). We first investigated the riboflavin‐mediated photocatalytic oxidation of the nucleotides and the generation of H_2_O_2_, under irradiation by blue light. In the presence of horseradish peroxidase (HRP), H_2_O_2_ oxidizes the 3,3′,5,5′‐tetramethyl benzidine (TMB) to form the colorimetric charge transfer complex with the maximum absorbance at 652 nm. The amount of H_2_O_2_ can be quantified by monitoring the absorbance changes at 652 nm (Figure S2, standard curve, Supporting Information), and was found to be dependent on the irradiation time (Figure 2B; Figure S3, Supporting Information), which was determined to be 10 min for the subsequent experiments. Figure S4 (Supporting Information) shows that among different guanine‐derived nucleotides, the oxidation of guanosine generated the highest amount of H_2_O_2_, probably because of the hydrogen bonding and stacking interactions between guanosine and riboflavin. It was also interesting to find that the oxidation of guanosine led to significantly more H_2_O_2_ than adenosine, uridine, and cytidine (Figure 2C; Figure S5, Supporting Information), which is attributed to much lower potential of guanosine than other nucleotides (*E°* vs NHE = 1.29 V for guanosine, 1.42 V for adenosine, 1.6 V for cytidine, and 2.4 V for uridine).^[^ ^29^, ^30^ ^]^ The dependence of H_2_O_2_ amount on the nucleobase species was further confirmed by using nucleotide monophosphates (GMP, CMP, AMP, or UMP) as the reductants (Figure S6, Supporting Information). The oxidation products of guanosine were studied by electrospray mass spectrometry (Figure 2E–G). It was observed the imidazolone (*m*/*z*: 284.15), the oxazolone (hydrolytic products of imidazolone, *m*/*z*: 263.13), and an intermediate (*m*/*z*: 290.20), indicating that the reaction path followed the mechanism of the one‐electron oxidation of the guanosine as proposed in Figure S7 in the Supporting Information.^[^ ^31^ ^]^ In brief, the guanosine was abstracted an electron to generate the radical cation G^·+^, which was deprotonated and reacted with another superoxide anion radical, along with protonation. This process led to the generation of the hydroperoxide adduct at C5 of guanine, which can convert to imidazolone.^[^ ^32^, ^33^ ^]^ This process involves intramolecular nucleophilic attacks, accompanied by the release of carbon dioxide and formamide. When we replaced riboflavin with flavin mononucleotide (FMN) or flavin adenine dinucleotide (FAD), it is interesting to find that there was much less H_2_O_2_ generated (Figure 2D; Figure S8, Supporting Information), in particular for FAD as the photocatalyst, probably due to the intramolecular electron transfer from the excited flavin to adenine.^[^ ^34^ ^]^ Taken together these results, guanosine and riboflavin were selected as the components for constructing the supramolecular photocatalytic materials for H_2_O_2_ production.

**Figure 3:**
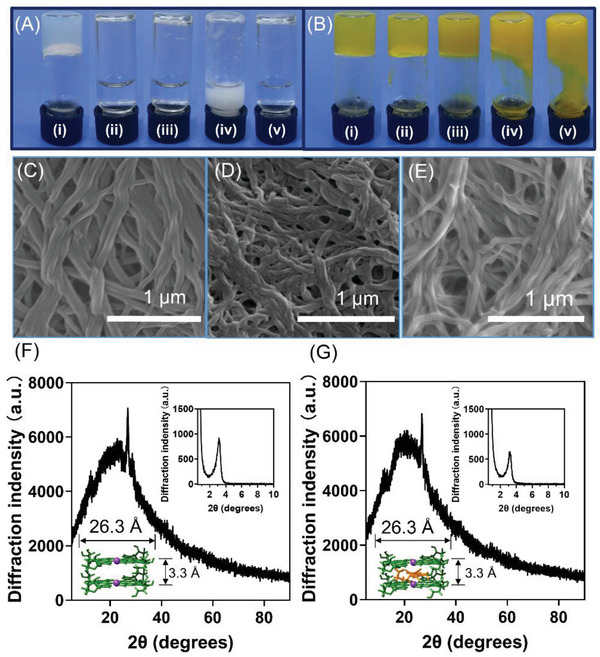
A) The images of the supramolecular G4‐samples self‐assembled from (i) guanosine/4‐FPBA/1,8‐diaminooctane/KCl, (ii) guanosine/1,8‐diaminooctane/KCl, (iii) guanosine/4‐FPBA/1,8‐diaminooctane, (iv) guanosine/4‐FPBA/KCl, and (v) guanosine/4‐FPBA/1,8‐diaminooctane/NaCl. B) Riboflavin‐loaded supramolecular G4‐samples containing riboflavin of (i) 0.5 × 10^−3^ m, (ii) 1 × 10^−3^ m, (iii) 2 × 10^−3^ m, (iv) 3 × 10^−3^ m, and (v) 4 × 10^−3^ m. C–E) SEM images of the lyophilized powder from (C) G4‐hydrogels, (D) the riboflavin‐loaded G4‐hydrogels, and (E) the riboflavin‐loaded G4‐hydrogels after 10 min blue light irradiation. F,G) XRD spectra of lyophilized powder from G4‐hydrogels (F) and riboflavin‐loaded G4‐hydrogels (G). Insets are SAXS spectra of G4‐hydrogels and riboflavin‐loaded G4‐hydrogels, respectively.

Guanosine can self‐assemble into G4‐supramolecular materials in the presence of 4‐FPBA, 1,8‐diaminooctane and KCl at a molar ratio of 1:0.5:0.25 in ultrapure water. As shown in Figure 1, guanosine formed G‐quartet via hydrogen bonding and then stacked into nanofibers. Meanwhile, guanosine was coupled to 4‐FPBA via reaction of cis‐diol with boronic acid to form cyclic ester, and the 1,8‐diaminoocatane was conjugated to 4‐FPBA in a stoichiometric ratio of 1/2 via condensation between amino group and the aldehyde group, leading to the crosslinking of the G4 nanowire (Figure S9, Supporting Information). It is noted that in the absence of guanosine, 4‐FPBA, or 1,8‐ diaminoocatane, or by replacing K^+^ with Na^+^, the solution cannot be solidified (**Figure** **3**A). Fourier transform infrared (FTIR) spectra show the disappearance of –OH (3300–3600 cm^−1^), B–OH (1282 cm^−1^), and C=O (1662 cm^−1^), and appearance of B–OC (1013 cm^−1^) and C=N (1695 cm^−1^) (Figure S10, Supporting Information). The formation of C=N was further confirmed by ^1^H NMR spectra, which show the emergence of the –CH=N signals (*δ* = 8.3 ppm), and the weakened –CHO peaks (*δ* = 9.8 ppm) compared to 4‐FPBA (Figure S11, Supporting Information). These spectral results demonstrated the reactions that led to the formation of the G4 supramolecular materials. Many G4 hydrogels were constructed by forming the borate ester as the crosslinker, and the alkaline condition was required to transform B(OH)_3_ to B(OH)_4_.^[^ ^22^, ^23^ ^]^ In comparison, 4‐FPBA/1,8‐diaminooctane/4‐FPBA conjugate has –B(OH)_2_ at both terminals, each of which can react with the cis‐diol of guanosine or riboflavin at a physiological pH, suitable for the biological application of this hydrogel.

**Figure 4:**
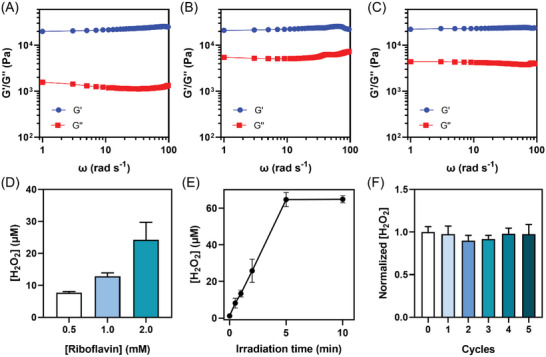
The rheological curve of A) G4‐hydrogel, B) non‐irradiated riboflavin‐loaded G4‐hydrogel and (C) post‐irradiated riboflavin‐loaded G4‐hydrogel. D) The amount of H_2_O_2_ generated from post‐irradiated G4‐hydrogels loading riboflavin of different concentrations (*N* = 3, data are shown as the mean ± standard deviation). [HRP]: 10 × 10^−9^ m, [TMB]: 0.3 × 10^−3^ m, [PBS]: 20 × 10^−3^ m, pH 7.4. Irradiation time: 10 min. E) The amount of H_2_O_2_ generated from post‐irradiated riboflavin‐loaded G4‐hydrogel with different irradiation time (*N* = 3, data are shown as the mean ± standard deviation). [HRP]: 10 × 10^−9^ m, [TMB]: 0.3 × 10^−3^ m, [PBS]: 20 × 10^−3^ m, pH 7.4. F) Normalized concentration of H_2_O_2_ generated from post‐irradiated riboflavin‐loaded G4‐hydrogel after cycled heating/cooling treatments (*N* = 3, data are shown as the mean ± standard deviation). [HRP]: 10 × 10^−9^ m, [TMB]: 0.3 × 10^−3^ m, [PBS]: 20 × 10^−3^ m, pH 7.4. Irradiation time: 10 min.

The riboflavin was conjugated to 4‐FPBA in a similar way to guanosine, and there was *π*–*π* stacking interactions between riboflavin and guanosine, which allowed the incorporation of riboflavin to the supramolecular materials. When the riboflavin concentration exceeded 2 × 10^−3^ m, the complex cannot be solidified (Figure 3B). The formation of 4‐FPBA/riboflavin conjugate is indicated by the mass spectra (*m*/*z* values 489.16 and 507.16) (Figure S12, Supporting Information). The red shift in CD signals (214 nm, p–*π* transition^[^ ^35^ ^]^ of guanine of GMP (Figure S13, Supporting Information) and the decrease in fluorescence (*λ*_em_ 525 nm) of riboflavin (Figure S14, Supporting Information), which was attributed to the energy transfer, can reflect the aromatic stacking of riboflavin to G‐quartet. The scanning electron microscope (SEM) images of the lyophilized powder of the G4‐supramolecular materials and riboflavin‐loaded materials show the densely‐packed cross‐layered fibers in both materials, revealing the role of G4 wires in the materials formation (Figure 3C,D). We observed the characteristic Bragg diffraction peaks at 2*θ* values 26.5° in the diffraction of X‐rays (XRD) spectrum and 3.1° in the small‐angle X‐ray scattering (SAXS) spectrum, which indicated the G‐quartet distance (3.3 Å) and width (26.5 Å) (Figure 3F). The riboflavin‐loaded supramolecular materials showed similar XRD and SAXS spectra (Figure 3G), indicating that adding riboflavin did not change the assembly structure. These two materials both exhibited gel–sol temperature around 70 °C (Figure S15, Supporting Information), and higher storage modulus than loss modulus (**Figure** **4**A,B), which are characteristic rheological behaviors of the hydrogels.

In the riboflavin‐loaded hydrogel materials, guanosine acts as the essential building blocks, and also the fuels for the synthesis of H_2_O_2_. No external reductant is added for the catalytic reactions. After irradiation with blue light (460 nm), there was no observed collapse of the hydrogels, as shown in SEM images (Figure 3E), and alteration in the gel–sol temperature (Figure S15, Supporting Information) and the rheological property (Figure 4C) for the riboflavin‐loaded G4 hydrogel materials, suggesting the robustness of the photocatalytic materials. The amount of the generated H_2_O_2_ can be controlled by the riboflavin amount (Figure 4D) and irradiation time (Figure 4E; Figure S16, Supporting Information). In the presence of 2 × 10^−3^ m riboflavin and after irradiation of the hydrogels for 10 min, about 65 × 10^−6^ m H_2_O_2_ was detected in the buffer, which indicated a small amount of guanosine to be oxidized by the photoexcited riboflavin. This may be because the riboflavin was fixed to the G4 wire through chemical conjugation or stacking, and only oxidized guanosine that was proximal to riboflavin. This low‐yield guanosine oxidation may account for the retained gel‐like properties of the supramolecular materials after light irradiation. The photocatalytic materials also showed tolerance against the room‐temperature storage (Figure S17, Supporting Information) and temperature cycling (Figure 4F; Figure S18, Supporting Information). It was found that after stored in darkness for a month, or 5 cycles of heating/cooling (65–25 °C) treatments, there was almost no change in the amount of H_2_O_2_ upon irradiation. . The 4‐FPBA/1,8‐diaminooctane conjugate acted as a longer spacer than other crosslinkers,^[^ ^24^, ^25^, ^36^, ^37^ ^]^ which may facilitate the access of the O_2_ to the riboflavin.

**Figure 5:**
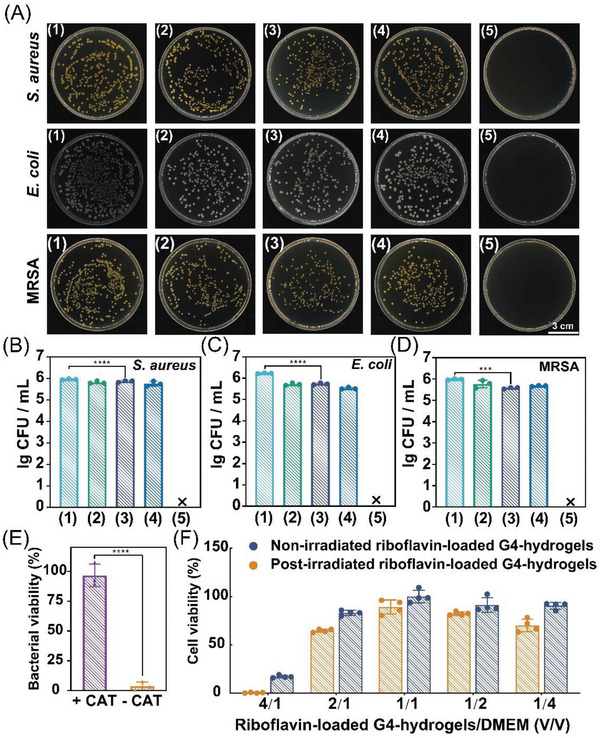
In vitro post‐irradiated antibacterial effect of the G4‐hydrogels. A) Bacterial colony images of *S. aureus*, *E. coli*, and MRSA after culture with (1) PBS, (2) non‐irradiated G4‐hydrogel, (3) non‐irradiated riboflavin‐loaded G4‐hydrogel, (4) post‐irradiated G4‐hydrogel, (5) post‐irradiated riboflavin‐loaded G4‐hydrogel. B–D) Colony forming units (CFU) of (B) *S. aureus*, (C) *E. coli*, (D) MRSA. E) Bacterial viability of the post‐irradiated riboflavin‐loaded G4‐hydrogel with or without catalase treatment (30 min). F) Cell viability in different relative concentration of riboflavin‐loaded G4‐hydrogel/Dulbecco's modified eagle medium (DMEM). *N* ≥ 3, data are shown as the mean ± standard deviation. *p < 0.05, **p < 0.01, ***p < 0.001, and ****p < 0.0001.

The in vitro antibacterial investigations were performed by coculturing the bacteria with the post‐irradiated riboflavin‐loaded G4‐hydrogels, in the tryptone soya agar (TSA). There is no observed bacterial growth in the TSA, as revealed by **Figure** **5**A. Figure 5B–D shows that the post‐irradiated riboflavin‐loaded hydrogels killed *S. aureus* (Gram‐positive bacteria), *E. coli* (Gram‐negative bacteria), and MRSA (multidrug resistant bacteria), with the sterilization ratio over 99.999%, which can be attributed to the generated H_2_O_2_. The photocatalytic generation of H_2_O_2_ and the antibacterial investigations were implemented in different environments. There was no difference in the amount of H_2_O_2_ exposed to different antibacterial groups. It is noteworthy that the non‐irradiation group also exhibited slight antibacterial activity, probably due to the bioactive riboflavin.^[^ ^38^ ^]^ To confirm the role of the H_2_O_2_, we incubated catalase with the post‐irradiated riboflavin‐load hydrogel for 30 min prior to cocultured with the bacteria, and found that the antibacterial activity of the hydrogel was completely inhibited (Figure 5E; Figure S19, Supporting Information). To test the biosafety of the photocatalytic system, we incubated the post‐irradiated riboflavin‐loaded G4‐hydrogel with Dulbecco's modified eagle medium (DMEM) which contained L929 cell with identical concentration to the bacteria. The cell viability was over 80% (Figure 5F), indicating the produced H_2_O_2_ exhibited no significant cytotoxicity to the L929 cell.

**Figure 6:**
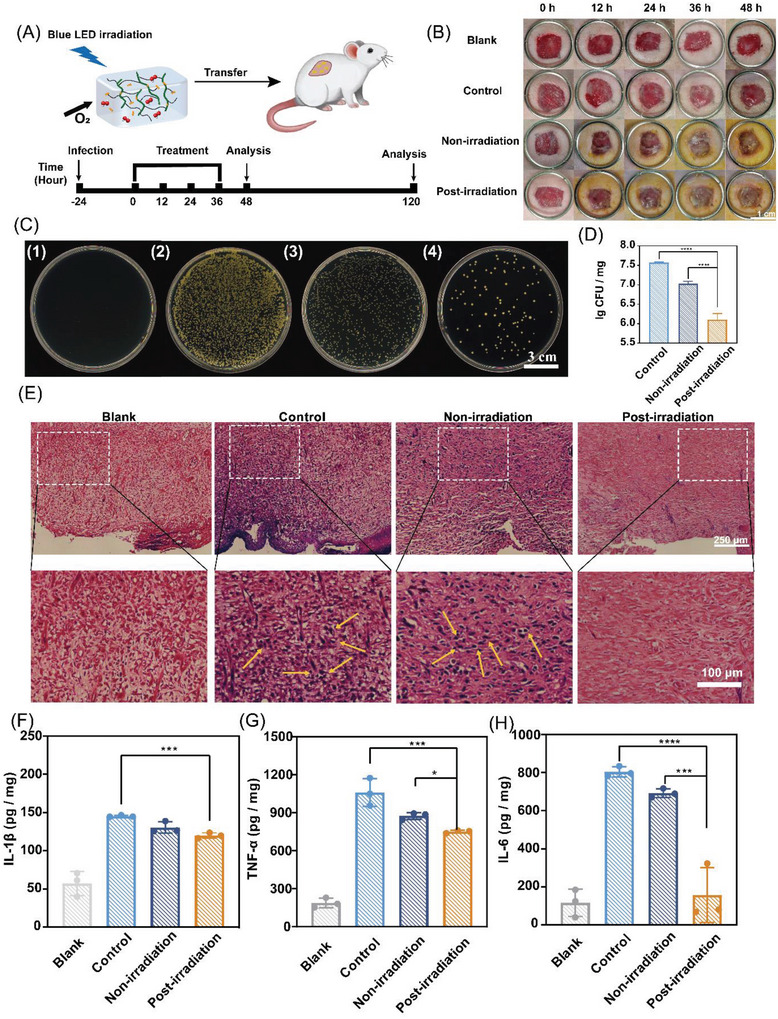
In vivo post‐irradiated antibacterial therapy. A) Schematic illustration of back wound infection treatment in rats. B) The wound area images of rats in different group at different times. C) Colony images of wound tissue dilution at 48 h. (1) Blank group; (2) Control group; (3) Non‐irradiation group; (4) Post‐irradiation group. D) CFU of wound tissue homogenate dilution with different treatments at 48 h. E) Images of Gram stained sections with different treatment at 48 h (Yellow arrows point to the MRSA). F–H) The concentrations of (F) IL‐1*β*, (G) TNF‐*α*, (H) IL‐6 at 48 h. *N* = 3, data are shown as the mean ± standard deviation. *p < 0.05, **p < 0.01, ***p < 0.001 and ****p < 0.0001.

The in vivo antibacterial activities were assayed by adding the post‐irradiated hydrogel dressing to the wound. A model of rat wound infected by MRSA was established to evaluate the therapeutic effect of the photocatalytic riboflavin‐loaded G4‐hydrogel. The rats were divided into four groups: a blank group without any wound infection treatment (Blank group); a control group with wound infection but without adding riboflavin‐loaded hydrogels (Control group); a non‐irradiated riboflavin‐loaded hydrogel group (Non‐irradiation group) and a post‐irradiated riboflavin‐loaded hydrogel treatment group (Post‐irradiation group). The wound infection treatment in rat was schematically illustrated in **Figure** **6**A. Figure 6B and Figure S20 (Supporting Information) shows the changes in wound morphology on the rats in different group. As revealed by Figure 6C,D, the wound tissue homogenate dilutions of the post‐irradiation group had the lower number of colonies than other groups, after treatment for 48 h, indicating higher capability of post‐irradiation group in killing bacteria in the wound, as a result of the H_2_O_2_ production. Interestingly, the non‐irradiation group also showed slight bactericidal activity, which is consistent with in vitro antimicrobial assays. The images of Gram‐stained sections of the wound tissue show that the bacteria count of the wound tissue in the post‐irradiation group was similar to that of the blank group (Figure 6E), confirming the bacteria was effectively killed by the post‐irradiated hydrogel. Subsequently, the levels of typical inflammatory factors in wound tissue homogenates, such as tumor necrosis factor‐*α* (TNF‐*α*), interleukin‐1*β* (IL‐1*β*), or interleukin‐6 (IL‐6), were measured, to further evaluate the therapeutic effect. The level of these factors was reduced in the post‐irradiation group (Figure 6F–H), which was also found in rats sacrificed at 120 h (Figure S21, Supporting Information). In hematoxylin‐eosin (H&E) stained sections at 120 h (Figure S22, Supporting Information), the inflammatory cells in the post‐irradiation group were almost the same to the wound tissue of the blank group. In addition, there was no significant change in the body weight of the rats during the treatment (Figure S23, Supporting Information). H&E staining sections and body weight results indicate that this post‐irradiation therapy did not affect the normal growth of the rats and had no obvious cytotoxicity.

## 3 Conclusion

In summary, we have presented the supramolecular riboflavin‐loaded G4 materials, which exhibited gel‐like properties, for the proof‐of‐concept post‐irradiation antibacterial therapy of the infected wound. The G4‐hydrogels served as the dressing materials to scaffold the riboflavin through covalent bonding and aromatic stacking, and provided the reductant guanosine for the reduction of the photoexcited riboflavin, followed by O_2_ reduction to generate H_2_O_2_. The post‐irradiated hydrogels exhibited high‐efficacy antibacterial activity toward killing Gram‐positive bacteria, Gram‐negative bacteria and multidrug resistant bacteria in vitro and in vivo, and showed biosafety and no obvious cytotoxicity. Unlike the traditional PDT that requires real‐time irradiation, the post‐irradiation therapy presented by this work relies on H_2_O_2_ that has considerably longer lifetime than the high‐reactivity ∙OH or singlet oxygen, etc. Therefore, there is no requirement for confinement of the patients to the illumination source, which may improve the compliance of the treatment. It is also noteworthy that these ∙OH or singlet oxygen species, which may be generated during the photo‐irradiation of riboflavin,^[^ ^28^ ^]^ were not involved in the antibacterial therapy, since there was ≈2 min interval when the post‐irradiated hydrogel was transferred to the infectious site of the rats. Our findings may provide a new insight into the photodynamic therapy.

## 4 Experimental Section

### Materials

Guanosine, adenosine, cytidine, uridine, guanine, GMP, AMP, CMP, UMP, GDP, GTP, H_2_O_2_, HRP, TMB, riboflavin, FMN, FAD, KCl, and NaCl were purchased from Aladdin. 4‐FPBA was purchased from Innochem. 1,8‐diaminooctane was purchased from Macklin. *E. coli* (ATCC 25 922), *S. aureus* (CMCC(B) 26 003), methicillin‐resistant *S. aureus* (MRSA ATCC 43 300), L929 cell lines were obtained from American Type Culture Collection (ATCC, Rockville, MD). Sprague–Dawley (SD) rats (Male, 6–7 weeks) were purchased from Beijing Vital River Laboratory Animal Technology Co., Ltd. (China). Rat interleukin‐1*β* enzyme‐linked immunosorbent assay kits (Chuangshi, ER09919‐96t), Rat interleukin‐6 enzyme‐linked immunosorbent assay kits (Chuangshi, ER09906‐96t), Rat tumor necrosis factor‐*α* enzyme‐linked immunosorbent assay kits (Chuangshi, ER09108‐96t) were purchased form Zancheng (Tianjin) Technology Co., Ltd. (China). Tryptone soya agar, yeast extract powder, tryptone were purchased from Beijing Aoboxing Bio‐Tech Co., Ltd. Sodium chloride was purchased from Fuchen (Tianjin) chemical reagent Co., Ltd. Cell viability assays was obtained using cell imaging multimode reader (BioTek Instruments, Cytation 3).Ultrapure water was deionized by a Milli‐Q system.

### Acquisition of Standard Curves

HRP was added into PBS buffer (pH 7.4) and mix to homogeneous solution. With TMB (*ε*: 39 800 m ^−1^cm^−1^) and H_2_O_2_ as the substrates, the time‐dependent absorbance changes were recorded, which were used to calculate the initial catalytic velocity by Lambert–Beer law (*V_i_*). The UV–vis absorption spectra were recorded using UV‐2600 spectrometer equipped with temperature controlling accessory (Shimadzu, Japan).

In the presence of 10 × 10^−9^ m HRP, plotted *V_i_* against the concentration of the H_2_O_2_ while the concentration of TMB was 0.3 × 10^−3^ m. Then, the global fitting approach was used to evaluate the turnover frequency (*k*_cat_), Michalis–Menten constants (*K*_m_). The fitted curve reflects the relationship between the concentration of H_2_O_2_ and *V_i_* of TMB oxidation (Figure S2, Supporting Information). The concentration of H_2_O_2_ can be calculated from this curve using the following formula

(1) $$\left[H_{2}O_{2}\right]=V_{i}\times 122.7/\left(2695-V_{i}\right)$$

### Quantification of H_2_O_2_ Generated by Riboflavin Mediated Photo‐oxidation of Guanosine in Solution

Guanosine and riboflavin were added to ultrapure water and irradiated by 460 nm light. After that, added HRP and TMB to the solution and recorded the time‐dependent absorbance changes. *V_i_* was calculated by Lambert–Beer law and the concentration of H_2_O_2_ was calculated using standard curve. All measurement repeated for three times.

### Mass Spectra

The mass spectra were recorded using Bio‐LCMS8050 mass‐spectrometer (Shimadzu, Japan) using an ESI (electro‐spray‐ionization) source. The samples were dissolved in ultrapure water and equal volume of acetonitrile was added. The spectra were recorded in the positive ion mode.

### Preparation of G4‐ and Riboflavin‐Loaded G4‐Hydrogel

For preparing G4‐suramolecular hydrogel, 35 × 10^−3^ m guanosine, 35 × 10^−3^ m 4‐FPBA, 17.5 × 10^−3^ m 1.8‐diaminooctane, and 8.75 × 10^−3^ m KCl were added into ultrapure water and boiled to transparent solution. G4‐suramolecular hydrogel was formation after cooled down to room temperature. For loading riboflavin to G4‐hydrogel, riboflavin was added to the boiling transparent solution and continued to stir for one minute. Then cooled it to room temperature and solid riboflavin‐loaded G4‐hydrogel was formation.

### Characterization of G4‐ and Riboflavin‐Loaded G4‐Hydrogel

G4‐ and riboflavin‐loaded G4‐hydrogel were lyophilized into powder for FTIR, XRD, SAXS, and SEM characters. For FTIR, the lyophilized powder of G4‐ or riboflavin‐loaded G4‐hydrogel were mixed with KBr at 1:100, and then the spectra were recorded by Tensor II spectrometer (Bruker, America) with the scan frequency at 7.5 KHz. The XRD spectra were recorded using Ultima IV X‐ray diffractometer (Rigaku, Japan). The SAXS spectra were recorded using Xuess2‐0 X‐ray diffractometer (Xenocs, France). For SEM, the samples were sputter‐coated with gold, the SEM morphological images were recorded using JSM‐7800F scanning electron microscope (JEOL, Japan).

G4‐hydrogel was prepared in D_2_O for 1H NMR characters. The ^1^H NMR spectra were recorded by 400 MHz AVANCE III spectrometer (Bruker, America), keep the sample in a transparent solution state during the measurement.

For rheology measurement, the G4‐ or riboflavin‐loaded G4‐hydrogel samples were prepared on the circle plates with 50 mm in diameter. The rheology curves were recorded by MCR102 rotational rheometer (Anton Parr, Austria). The rheology behavior of samples were analyzed by performing frequency sweep analysis over an angular frequency (*ω*) of 100–0.1 rad s^−1^.

The gel–sol temperature of G4‐ or riboflavin‐loaded G4‐hydrogel were obtained as follow steps: the samples were first heated to a specific temperature and then reversed the sample bottle and stayed for 5 min. If the samples did not collapse, continue to increase the temperature and repeat the steps until the samples collapsed, at which point the temperature was the gel–sol temperature. All measurements were repeated three times.

### Circular Dichroism (CD)

The CD spectra were measured by a J‐815 spectropolarimeter (Jasco, Japan), and the instrument needs to be supplied with nitrogen at a certain flow rate during the measurement. Set the parameters with bandwidth of 10 nm, scan speed of 100 nm min^−1^, standard sensitivity. Set PBS (20 × 10^−3^ m, pH 7.4) buffer as baseline and 400 µL sample was added into cuvette for measurement. All measurements were repeated three times.

### Fluorescence Spectrum

Fluorescent emission spectra were recorded using a G9800A fluorescence spectrophotometer with a temperature‐control accessory (Agilent, America). Riboflavin or riboflavin/GMP samples were added in PBS (20 × 10^−3^ m, pH 7.4) buffer. Set the slit width to 10 nm. The excitation wavelength was set to 450 nm and the emission spectra of 500–600 nm were collected.

### Quantification of H_2_O_2_ Generated by Post‐irradiated Riboflavin‐Loaded G4‐Hydrogel

PBS buffer was added on the surface of solid riboflavin‐loaded G4‐hydrogel and irradiated for 10 min. 200×10^‐6^ L PBS for quantificaiton of H_2_O_2_ generated from post‐irradiated G4‐hydrogels loading different concentration of riboflavin; 100×10^‐6^ L PBS for quantification of H_2_O_2_ generated from post‐irradiated riboflavin‐loaded G4‐hydrogel with different irradiation time. After that, the concentration of H_2_O_2_ in the PBS (20 × 10^−3^ m, pH 7.4) buffer was quantification by the same method as that in guanosine/riboflavin solution. All measurement repeated for three times.

### In Vitro Antibacterial Assay

Prepare Luria–Bertani (LB) medium according to 0.5 g NaCl, 1 g tryptone, 0.5 g yeast extract powder per 100 mL deionized water and autoclave. Prepare Tryptone soya agar (TSA) medium according to the instructions. First, the bacteria were cultured in LB medium at 37 °C overnight to form a final density of 1×10^8^ colony forming units (CFU) mL^−1^. The bacteria in LB medium were diluted to 2×10^5^ CFU mL^−1^ in PBS buffer. Prepare 100 µL of G4‐ or riboflavin‐loaded G4‐hydrogel in a 96‐well plate and add 50 µL of PBS buffer to per well. G4‐ and riboflavin‐loaded G4‐hydrogel requiring light irradiation were exposure in blue light at a wavelength of 460 nm and an optical power density of 80 mW cm^−2^ for 10 min. The bacterial solution (50 µL) was added to per well and incubated at 37 °C for 3 h. Afterwards, the bacterial solution (10 µL) was diluted 100‐fold and took 50 µL to disperse in solid TSA medium and incubated at 37 °C for 18 h and the number of colonies was counted.

### Hydrogen Peroxide Sterilization Verification Assay

Dissolve the catalase in PBS buffer solution at a concentration of 10 × 10^−6^ m. G4‐hydrogel (100 µL) was prepared in a 96‐well plate riboflavin‐loaded G4‐hydrogel was irradiated with blue light at a wavelength of 460 nm and an optical power density of 80 mW cm^−2^ for 10 min. Add 50 µL of PBS buffer to per well. For +CAT group, adding 25 µL of 10 × 10^−6^ m catalase solution per well and incubate at 37 °C for 30 min. For ‐CAT group, adding 25 µL of PBS buffer per well. The bacteria in LB medium were diluted with PBS to 4×10^5^ CFU mL^−1^. The bacterial solution (25 µL) was added to per well. The bacterial solution (25 µL) and 10 × 10^−6^ m catalase solution (25 µL) were added in PBS buffer (50 µL) was used as a blank set. After 3 h incubation, the bacterial solution (10 µL) was diluted 100‐fold and took 50 µL to disperse in solid TSA medium and incubated at 37 °C for 18 h, and the number of colonies was counted.

### In Vitro Cell Activity Assay

L929 cells were selected to determine the cytotoxicity of riboflavin‐loaded G4‐hydrogel by MTT assay. The riboflavin‐loaded G4‐hydrogel with irradiation group was irradiated with blue light at a wavelength of 460 nm and an optical power density of 80 mW cm^−2^ for 10 min. riboflavin‐loaded G4‐hydrogel was macerated with Dulbecco's modified eagle medium (DMEM) for 3 h and then the original extract was collected. Finally, different concentrations of the extracts were obtained by the doubling dilution to the original extract. Inoculate 1×10^4^ cells into each well of a 96‐well plate with 100 µL of DMEM medium (containing FBS and penicillin) and incubated under standard conditions. After 24 h of incubation, the medium was replaced with 100 µL of different concentrations of the extracts and incubated for 12 h. Cells cultured in sterile water and fresh DMEM medium were used as the corresponding positive and negative controls. Prepare MTT solution in advance, (5 mg MTT, 1 mL PBS, 9 mL DMEM) After aspiration of the original medium, 100 µL of MTT solution was added to incubate for 4 h and MTT solution was replaced with 100 µL of DMSO per well. The OD value at 570 nm was read by cell imaging multimode reader (BioTek Instruments, Cytation 3) and the cell viability was calculated.

### In Vivo Anti‐Infective Therapy

Animal studies were approved by the Ethical Committee of Chinese Academy of Medical Sciences (CAMS). All procedures were carried out in accordance with the Care and Use of Laboratory Animals (license number: 2022D019). A total of 24 rats were anesthetized with isoflurane and their backs were shaved, where two 1.5 × 1.5 cm^2^ full‐thickness skin wounds were created on their back skin by excision. Then the rats with wounds were randomly divided into four groups: Blank group (causing wounds only) Control group (no treatment after infection), non‐irradiation group (treatment by riboflavin‐loaded G4‐hydrogel after infection), Post‐irradiation group (treatment by riboflavin‐loaded G4‐hydrogel with irradiation after infection). Wounds in the blank group were treated with sterile dressing. Other groups were infected with 100 µL of 1×10^8^ MRSA solution for 24 h to establish the model. In Post‐irradiation group, the riboflavin‐loaded G4‐hydrogel (2 mL) was evenly applied to the sterile dressing to irradiated with blue light at a wavelength of 460 nm and an optical power density of 80 mW cm^−2^ for 10 min. Afterwards, riboflavin‐loaded G4‐hydrogel was fixed to the wound. In non‐irradiation group, the riboflavin‐loaded G4‐hydrogel (2 mL) was evenly applied to the sterile dressing and fixed to the wound. Wounds in the control group were treated with sterile gauze. Treatments were given every 12 h for a total of four sessions. Three rats were sacrificed respectively at 48 h and 120 h and the entire wound with adjacent normal skin was excised and fixed in 4% paraformaldehyde for hematoxylin‐eosin (H&E) staining and Gram staining. Infected tissues were isolated in PBS and homogenized and diluted 1000‐fold. A series of diluted samples were inoculated on TSA medium to count the growing colonies, which were used to analyze the number of bacteria in the infected tissue. CFU was calculated by the following formula

(2) CFU=9000×growingcolonies

Inflammatory factors of the wound tissue were determined according to the manufacturer's instructions of the ELISA kit.

### Statistical Analysis

Experiments were repeated at least three times and data presented as the mean ± standard deviation. All data were tested by Shapiro–Wilk approach for normal distribution. Differences between the two groups were analyzed by Student's *t*‐test. The differences among the three groups and above were analyzed by one‐way ANOVA analysis. The non‐parametric test was used, if the normal distribution was not satisfied. Sample size (*N*) for each statistical analysis was displayed in the corresponding figure. A *p* value < 0.05 (two‐sided) was considered statistically significant. Statistical significance was set at *p < 0.05, **p < 0.01, ***p < 0.001, and ****p < 0.0001. All analyses were carried out using GraphPad Prism 8.0.2 software

## Conflict of Interest

The authors declare no conflict of interest.

## Supporting information

Supporting Information

## Data Availability

The data that support the findings of this study are available from the corresponding author upon reasonable request.
