# Supplementary material for: A H2O2‐Supplied Supramolecular Material for Post‐irradiated Infected Wound Treatment
Source: Adv Sci (Weinh). 2023 Jan 29;10(9):2206851. doi: 10.1002/advs.202206851 (PMC10037955; doi:10.1002/advs.202206851)

ADVANCED SCIENCE Open Access

Supporting Information

for *Adv. Sci.*, DOI 10.1002/advs.202206851

A H_2O_2-Supplied Supramolecular Material for Post-irradiated Infected Wound Treatment

Peidong Du, Yanzhe Shen, Baoli Zhang, Shan Li, Minzheng Gao, Ting Wang, Xiaokang Ding, Bingran Yu, Zhen-Gang Wang* and Fu-Jian Xu**

Supporting Information

A H_2O_2-Supplied Supramolecular Material for Post-irradiated Infected Wound Treatment

Peidong Du,+ Yanzhe Shen,+ Baoli Zhang, Shan Li, Minzheng Gao, Ting Wang, Xiaokang Ding, Bingran Yu, Zhen-Gang Wang,* Fu-Jian Xu**

Mr. P. Du, Mr. Y. Shen, Prof. B. Yu, Prof. Z. G. Wang, Prof. F.J. Xu
State Key Laboratory of Organic-Inorganic Composites, Key Laboratory of Biomedical Materials of Natural Macromolecules (Beijing University of Chemical Technology, Ministry of Education), Beijing Laboratory of Biomedical Materials, Beijing University of Chemical Technology, Beijing, China

Ms. T. Wang, CAS Key Laboratory for Biomedical Effects of Nanomaterials and Nanosafety, National Center for Nanoscience and Technology, Beijing 100190, China.

E-mail: yubr@mail.buct.edu.cn; wangzg@mail.buct.edu.cn; xufj@mail.buct.edu.cn

Figure S1 UV-vis spectra of riboflavin in H_2O. ([Riboflavin]: 10 μM)

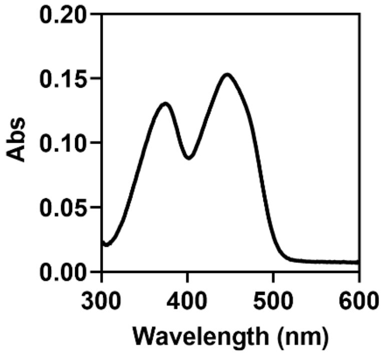

Figure S2 (A) Time dependent change of absorbance at 652 nm for H_2O_2 oxidation of TMB catalyzed by HRP. [TMB]: 0.3 mM, [HRP]: 10 nM, [PBS]: 20 mM, pH 7.4. (B) Global fitting of kinetic curve of H_2O_2 oxidization of TMB catalyzed by HRP. N = 3, data are shown as the mean ± standard deviation.

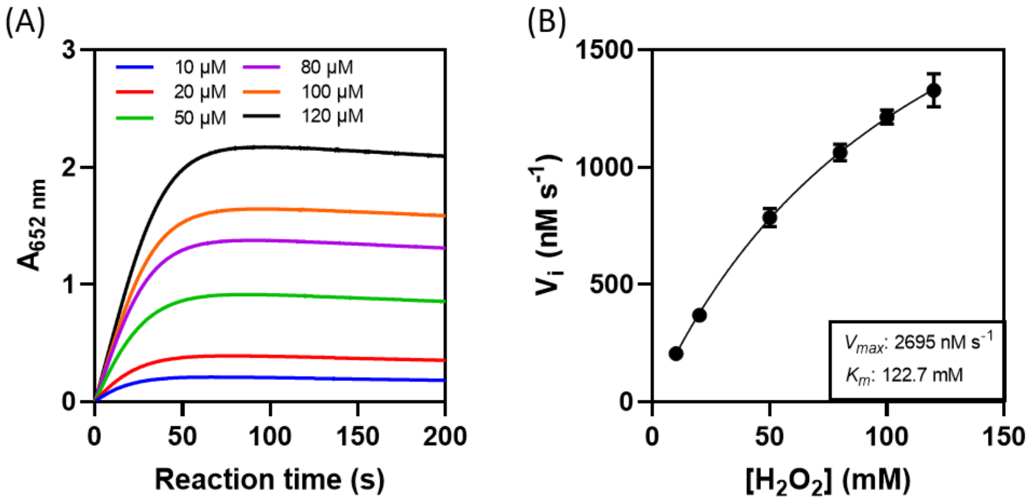

Figure S3 Time dependent change of absorbance at 652 nm for in situ produced H_2O_2 oxidation of TMB catalyzed by HRP. H_2O_2 was generated by riboflavin mediated photo-oxidation of guanosine with different irradiation time. (N = 3, data are shown as the mean ± standard deviation). [riboflavin]: 10 μM, [guanosine]: 200 μM, [HRP]: 10 nM, [TMB]: 0.3 mM, [PBS]: 20 mM, pH 7.4

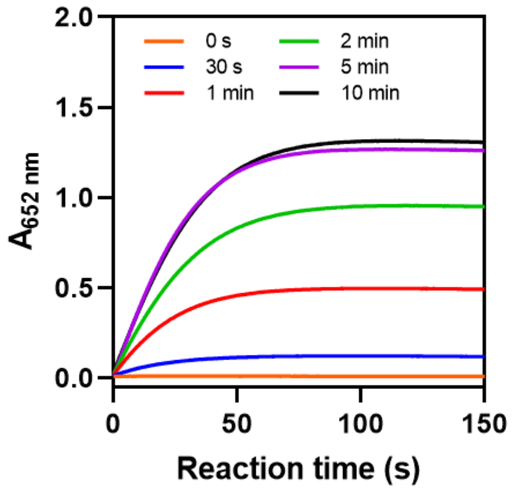

Figure S4 (A) Time dependent change of absorbance at 652 nm for H_2O_2 oxidation of TMB catalyzed by HRP. H_2O_2 was generated by riboflavin-mediated photo-oxidation of different guanine-derived nucleotides. (B) The amount of H_2O_2 generated by riboflavin mediated photo-oxidation of different guanine-derived nucleotides. (N = 3, data are shown as the mean ± standard deviation). Control group is riboflavin irradiated in the absence of the guanine-derived nucleotides. [riboflavin]: 10 μM, [substrates]: 200 μM, [HRP]: 10 nM, [TMB]: 0.3 mM, [PBS]: 20 mM, pH 7.4. Irradiation time: 10 min.

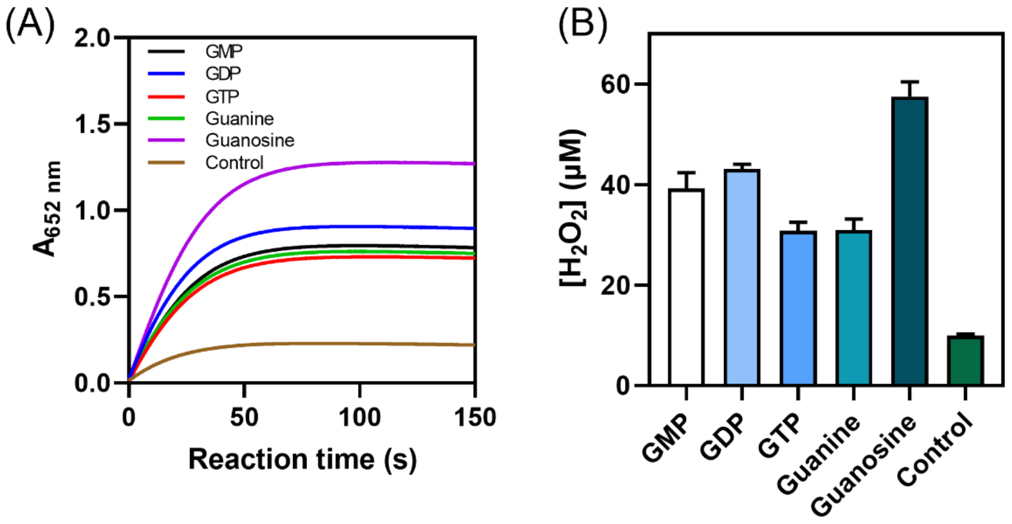

Figure S5 Time dependant change of absorbance at 652 nm for H_2O_2 oxidation of TMB catalyzed by HRP. H_2O_2 generated by riboflavin-mediated photo-oxidation of different nucleoside. [riboflavin]: 10 μM, [nucleoside]: 200 μM, [HRP]: 10 nM, [TMB]: 0.3 mM, [PBS]: 20 mM, pH 7.4. Irradiation time: 10 min.

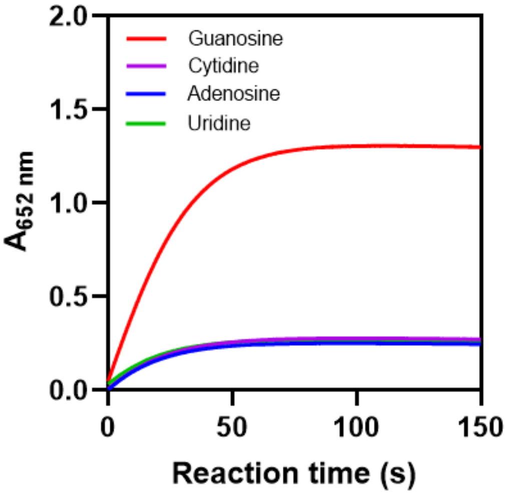

Figure S6 Evaluation of H_2O_2 production by photo-oxidation of GMP. (A) Time dependant change of absorbance at 652 nm for H_2O_2 oxidation of TMB catalyzed by HRP. H_2O_2 generated by riboflavin-mediated photo-oxidation of different nucleotide. (B) The amount of H_2O_2 generated by riboflavin mediated photo-oxidation of different nucleotide. (C) Time dependant change of absorbance at 652 nm for H_2O_2 oxidation of TMB catalyzed by HRP. H_2O_2 generated by riboflavin-mediated photo-oxidation of GMP with different irradiation time. (D) The amount of H_2O_2 generated by riboflavin mediated photo-oxidation of GMP with different irradiation time. [riboflavin]: 10 μM, [nucleotide]: 200 μM, [HRP]: 10 nM, [TMB]: 0.3 mM, [PBS]: 20 mM, pH 7.4. N = 3, data are shown as the mean ± standard deviation.

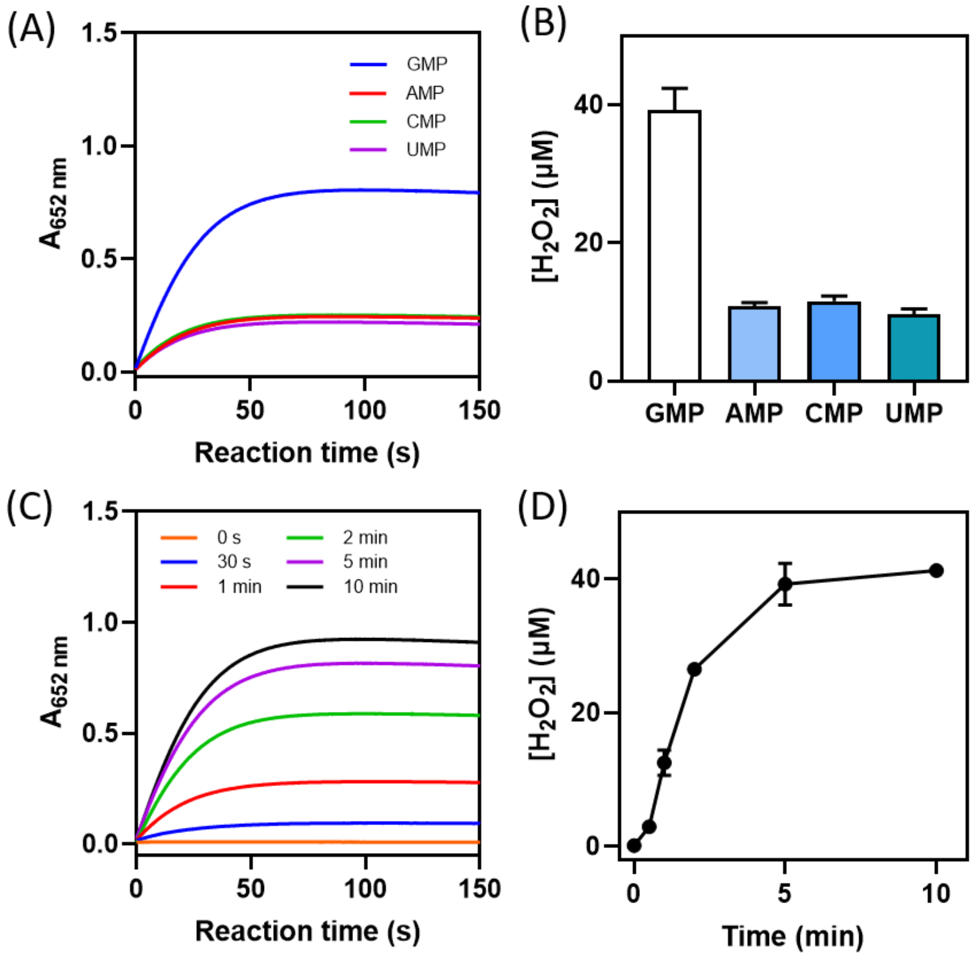

Figure S7 The process of photo-oxidation of guanosine in the presence of riboflavin.

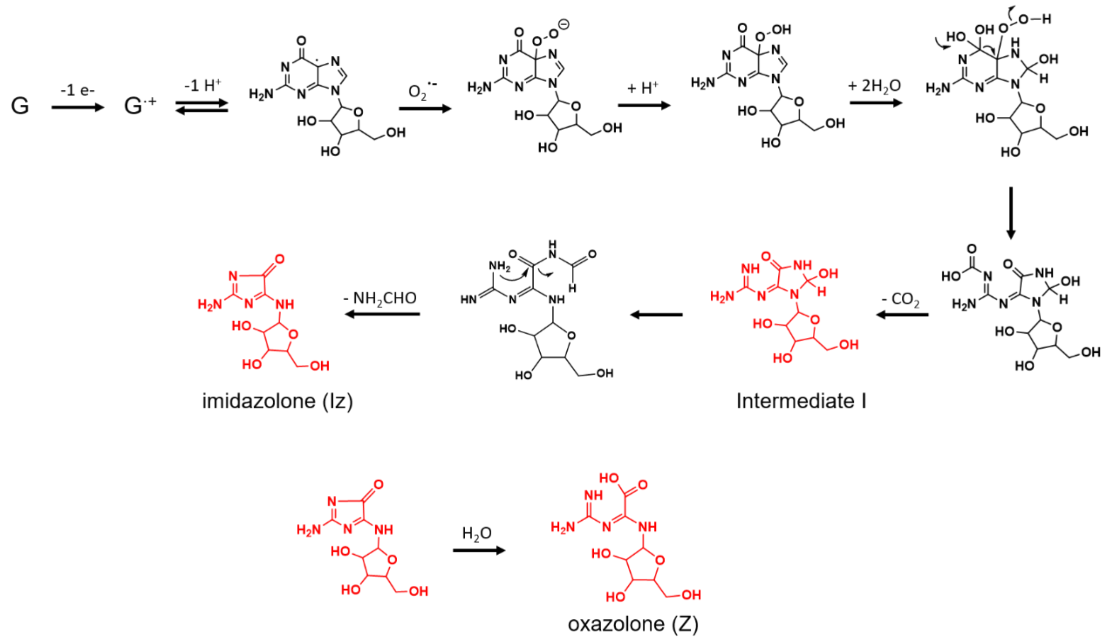

Figure S8 Time dependent change of absorbance at 652 nm for H_2O_2 oxidation of TMB catalyzed by HRP. H_2O_2 generated by different photosensitizers mediated photo-oxidation of guanosine. [Photosensitizer]: 10 μM, [HRP]: 10 nM, [TMB]: 0.3 mM, [PBS]: 20 mM, pH 7.4. Irradiation time: 10 min

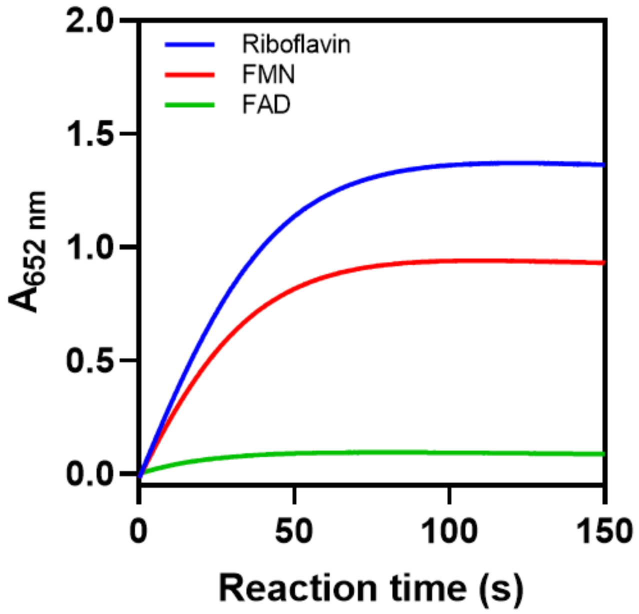

Figure S9 Proposed of the formation mechanism of G4-hydrogel. [1,2]

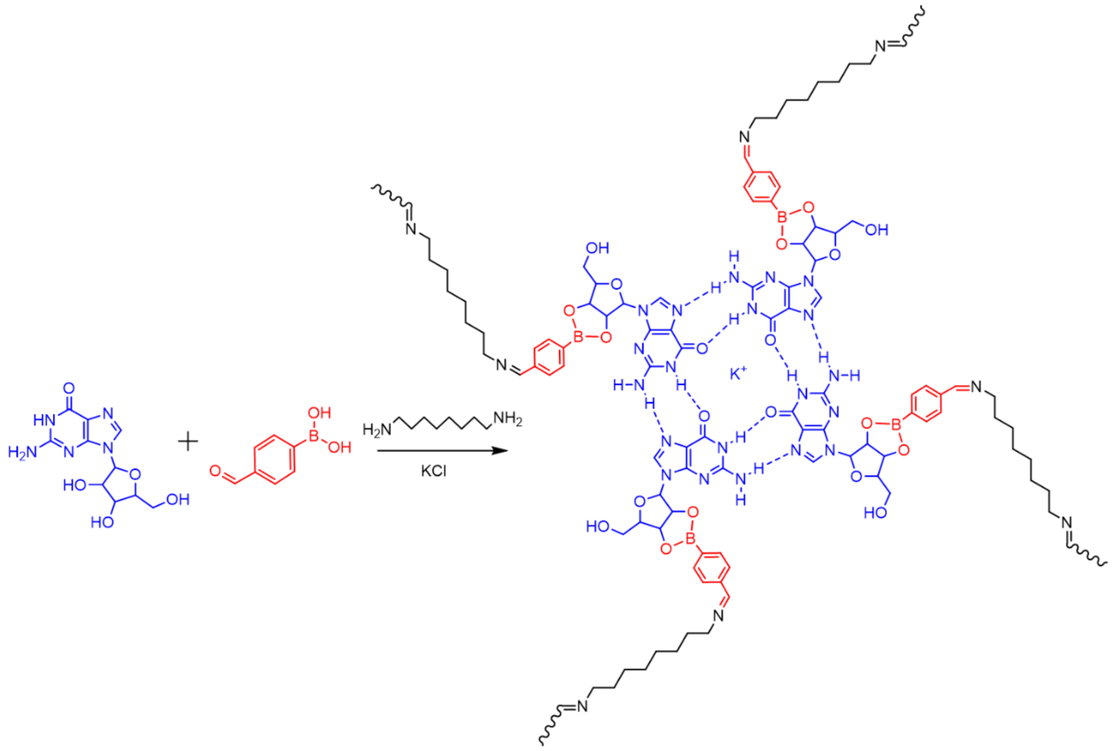

[1] Y. Li, Y. Liu, R. Ma, Y. Xu, Y. Zhang, B. Li, Y. An, L. Shi. *ACS Appl. Mater. Interfaces* 2017, **9**, 13056-13067.

[2] Y. Li, L. Su, Y. Zhang, Y. Liu, F. Huang, Y. Ren, Y. An, L. Shi, H. C. van der Mei, H. J. Busscher. *Adv. Sci.* 2022, **9**, 2103485.

Figure S10 FTIR spectra of guanosine, 4-FPBA and G4-hydrogel lyophilized powder.

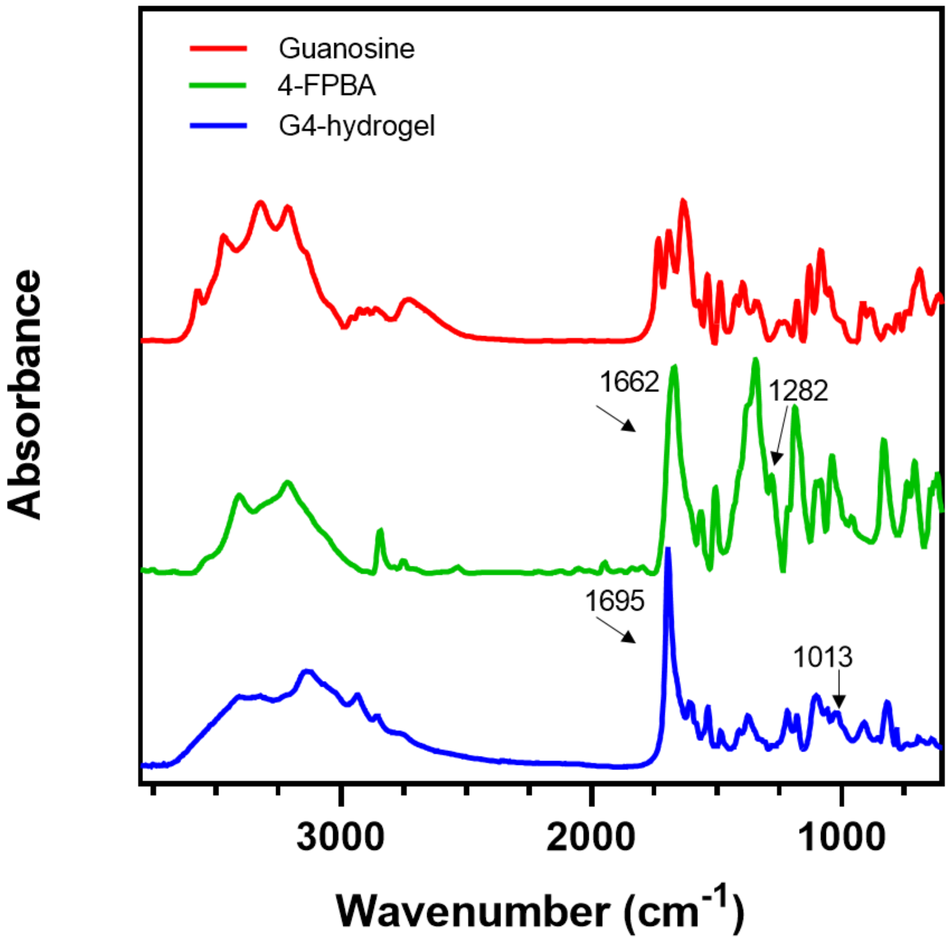

Figure S11 ^{1}H NMR spectra of guanosine, 4-FPBA, 1, 8-diaminoocatane and G4-hydrogel. Samples were dissolved in D_2O.

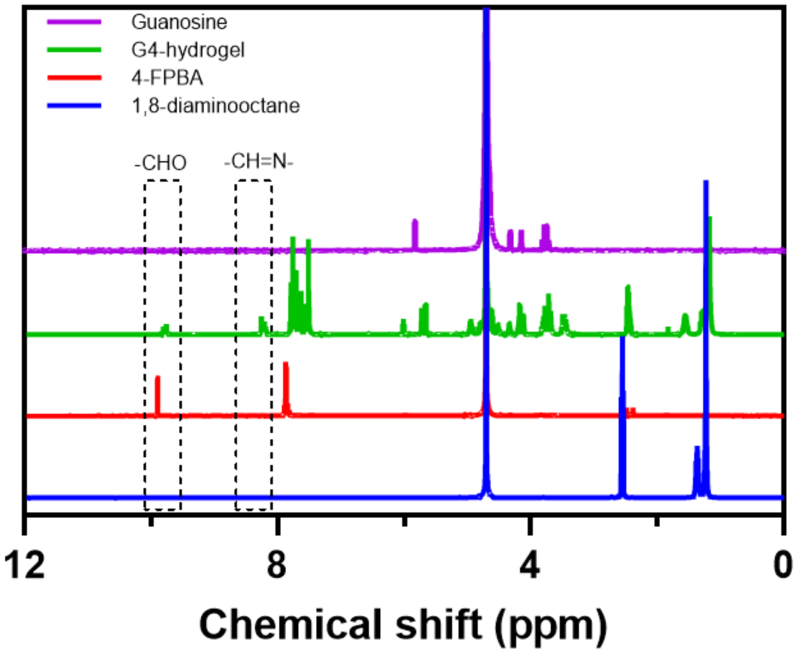

Figure S12 Mass spectra of (A) 4-FPBA, (B) riboflavin and (C-D) 4-FPBA/riboflavin. Figure D is the amplification of figure C with m/z range of 450-550. The mass spectra were recorded using an ESI (electro-spray-ionization) source and recorded in the positive ion mode.

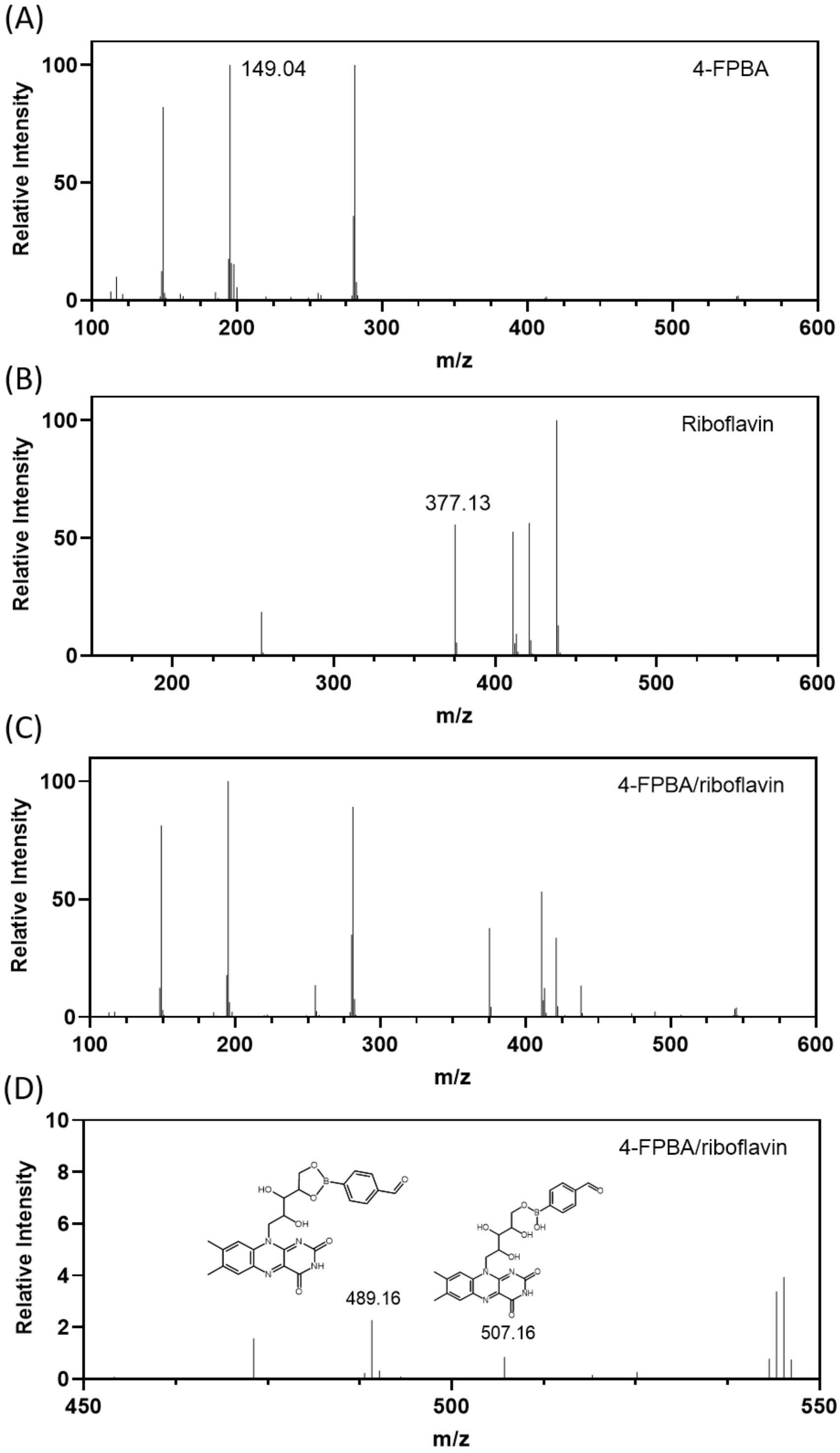

Figure S13 CD spectra of GMP and GMP/riboflavin complex. [GMP]: 2 mM.

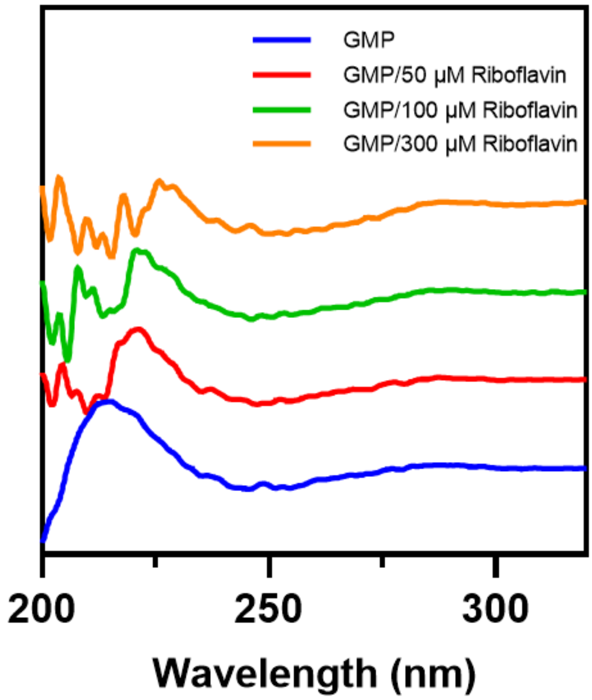

Figure S14 Fluorescence spectra of riboflavin and riboflavin/GMP. [Riboflavin]: 100 μM.

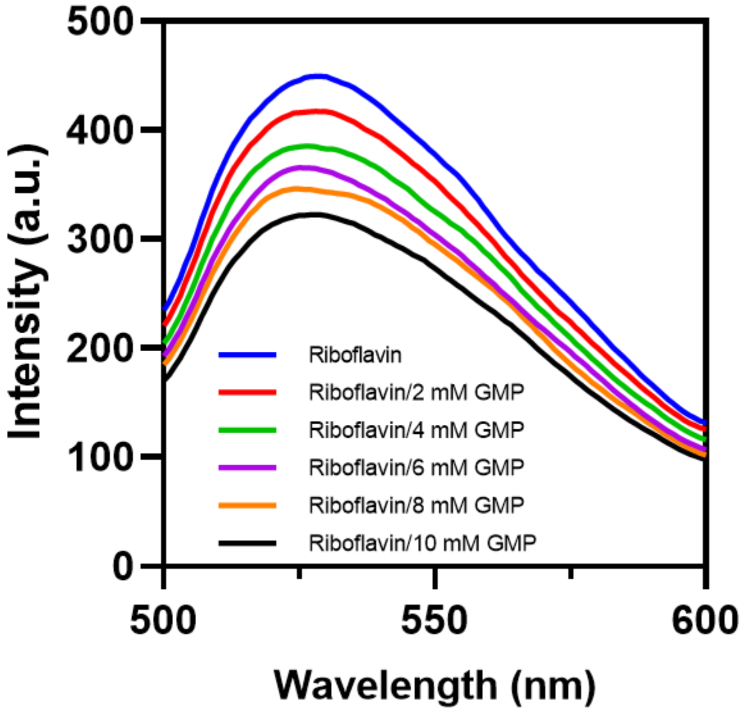

Figure S15 Gel-sol temperature of (A) G4-hydrogel, (B) non-irradiated riboflavin-loaded G4-hydrogel and (C) post-irradiated riboflavin-loaded G4-hydrogel (N = 3, data are shown as the mean ± standard deviation).

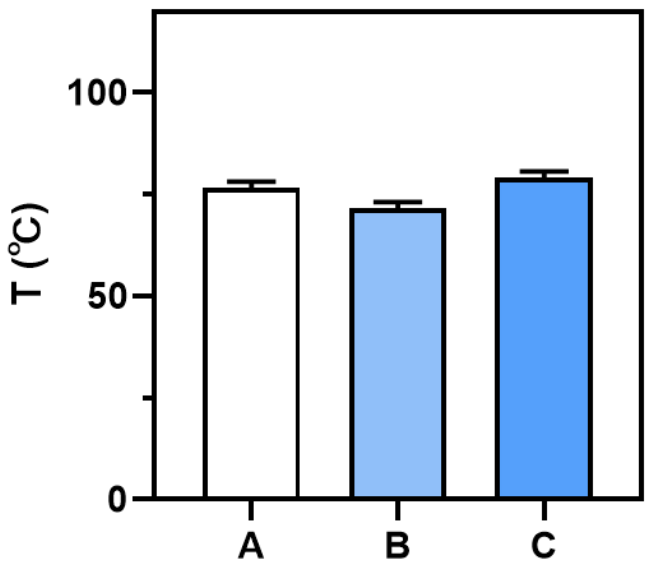

Figure S16 Time dependent change of absorbance at 652 nm for H_2O_2 oxidation of TMB catalyzed by HRP. H_2O_2 was generated from post-irradiated riboflavin-loaded G4-hydrogel with different irradiation time. (N = 3, data are shown as the mean ± standard deviation). [HRP]: 10 nM, [TMB]: 0.3 mM, [PBS]: 20 mM, pH 7.4.

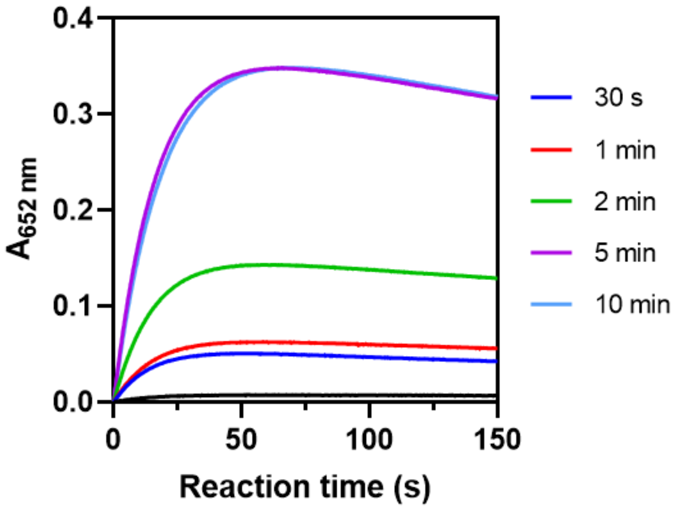

Figure S17 (A) Time dependent change of absorbance at 652 nm for H_2O_2 oxidation of TMB catalyzed by HRP. H_2O_2 was generated from riboflavin-loaded G4-hydrogel stored for different days with irradiation. (B) Normalized of H_2O_2 generated from post-irradiated riboflavin-loaded G4-hydrogel (N = 3, data are shown as the mean ± standard deviation). [HRP]: 10 nM, [TMB]: 0.3 mM, [PBS]: 20 mM, pH 7.4. Irradiation time: 10 min.

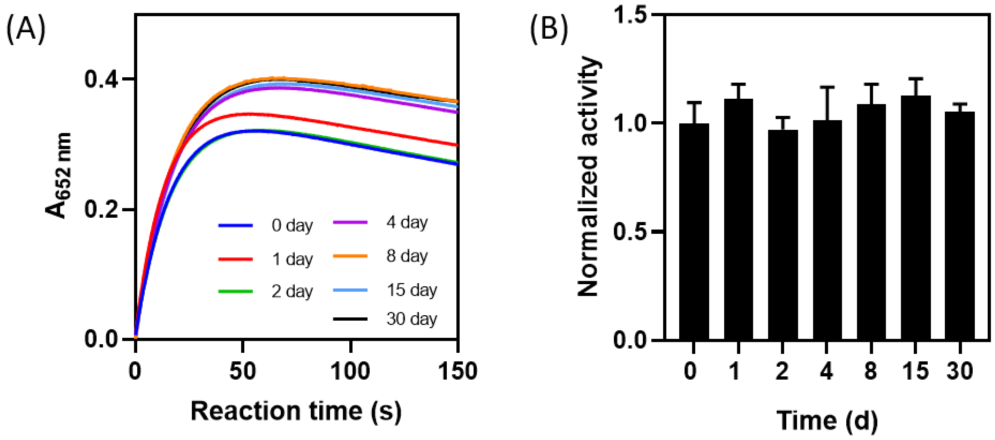

Figure S18 Time dependent change of absorbance at 652 nm for H_2O_2 oxidation of TMB catalyzed by HRP. H_2O_2 was generated from post-irradiated riboflavin-loaded G4-hydrogel. The number 0-6 represent the cycle time of heating/cooling treatment. (N = 3, data are shown as the mean ± standard deviation). [HRP]: 10 nM, [TMB]: 0.3 mM, [PBS]: 20 mM, pH 7.4. Irradiation time: 10 min.

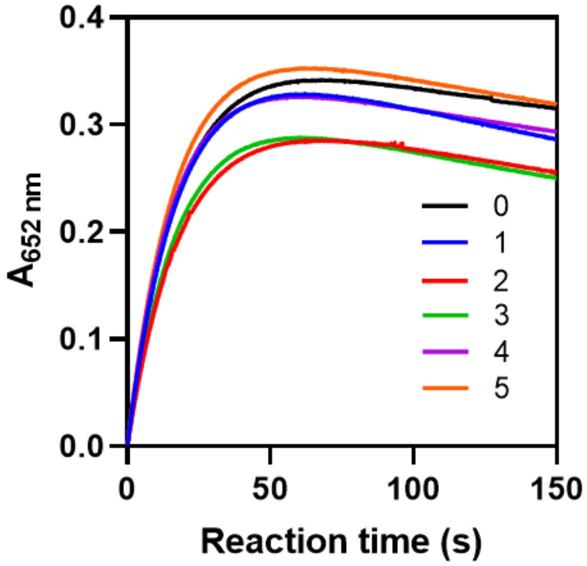

Figure S19 Bacterial colony images of bacterial solution cultured in post-irradiated riboflavin-loaded G4-hydrogel with incubating 2.0 mM catalase (CAT, 25 μL) for 30 minutes or without CAT.

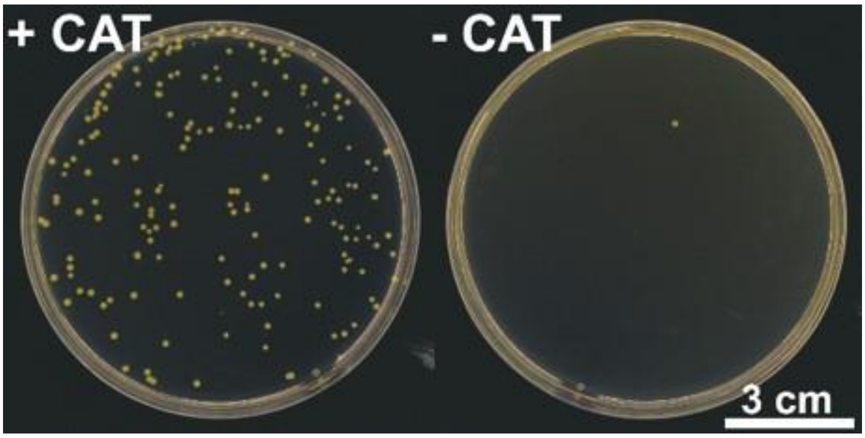

Figure S20 The wound area images of rats in different group at different times.

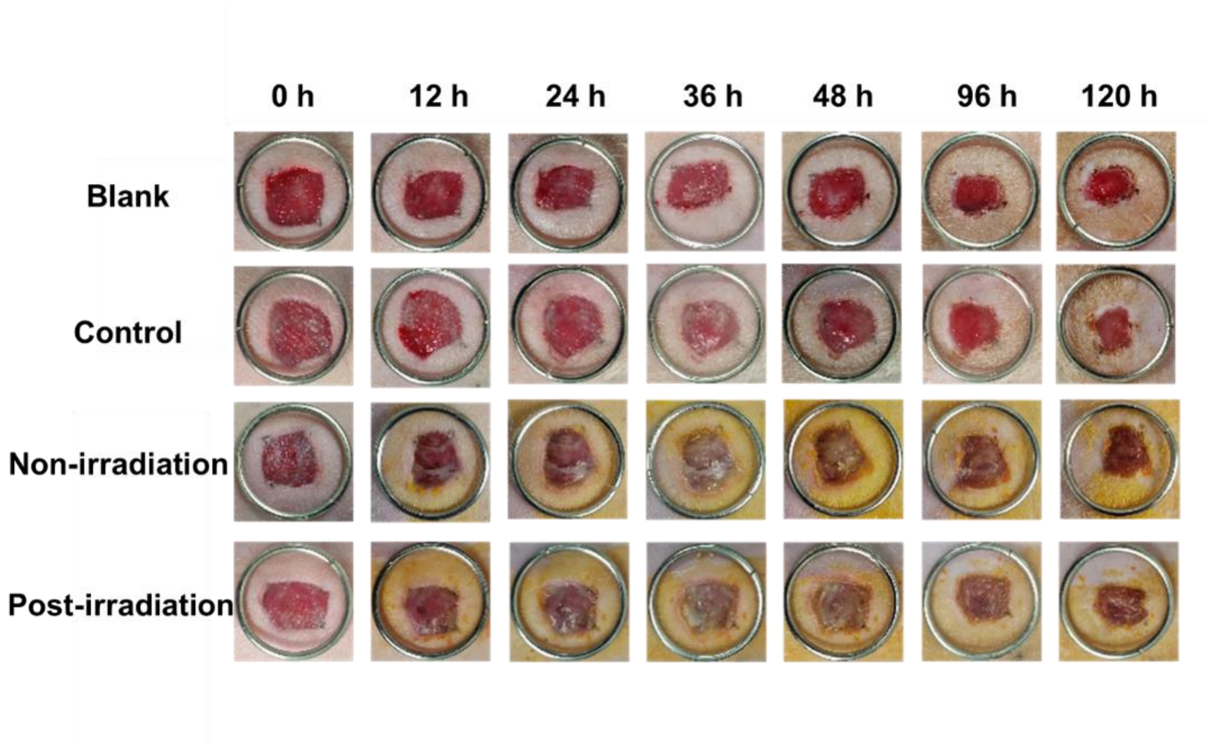

Figure S21 (A) Colony images of wound homogenates dilution at 120 h. Blank group (causing wounds only) Control group (no treatment after infection), Non-irradiation group (treatment by riboflavin-loaded G4-hydrogel after infection), Post-irradiation group (treatment by riboflavin-loaded G4-hydrogel with irradiation after infection). (B) Colony forming units (CFU) of different treatment at 120 h. (C) Images of Gram stained sections with different treatment at 120 h (Yellow arrows point to MRSA). (D)-(F) The concentrations of (D) interleukin-1β (IL-1β), (E) tumor necrosis factor-α (TNF-α), (F) interleukin-6 (IL-6) at 120 h. N = 3, data are shown as the mean ± standard deviation. *$p < 0.05$, **$p < 0.01$, ***$p < 0.001$ and ****$p<0.0001$.

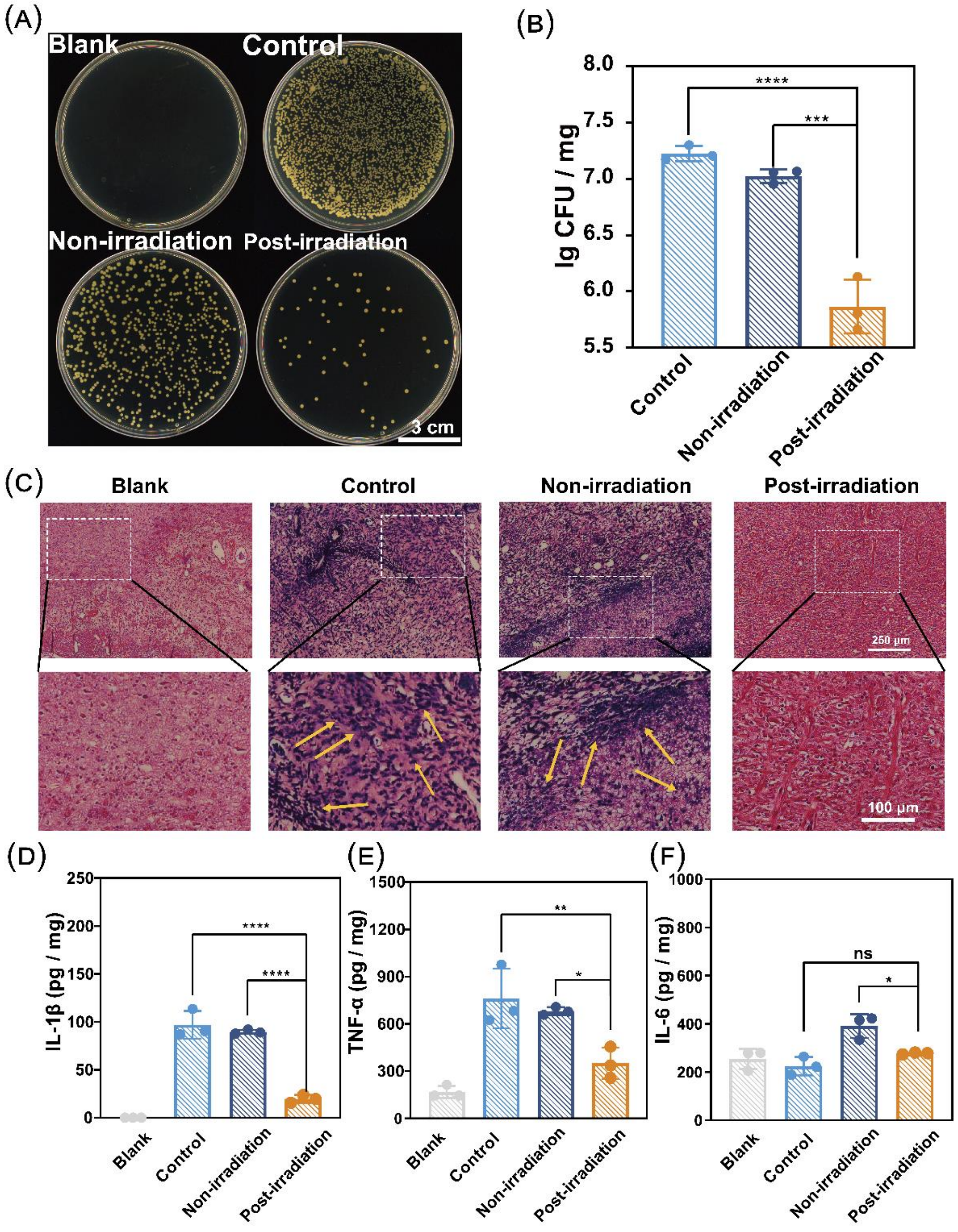

Figure S22 Images of H&E stained sections with different treatment at 120 h.

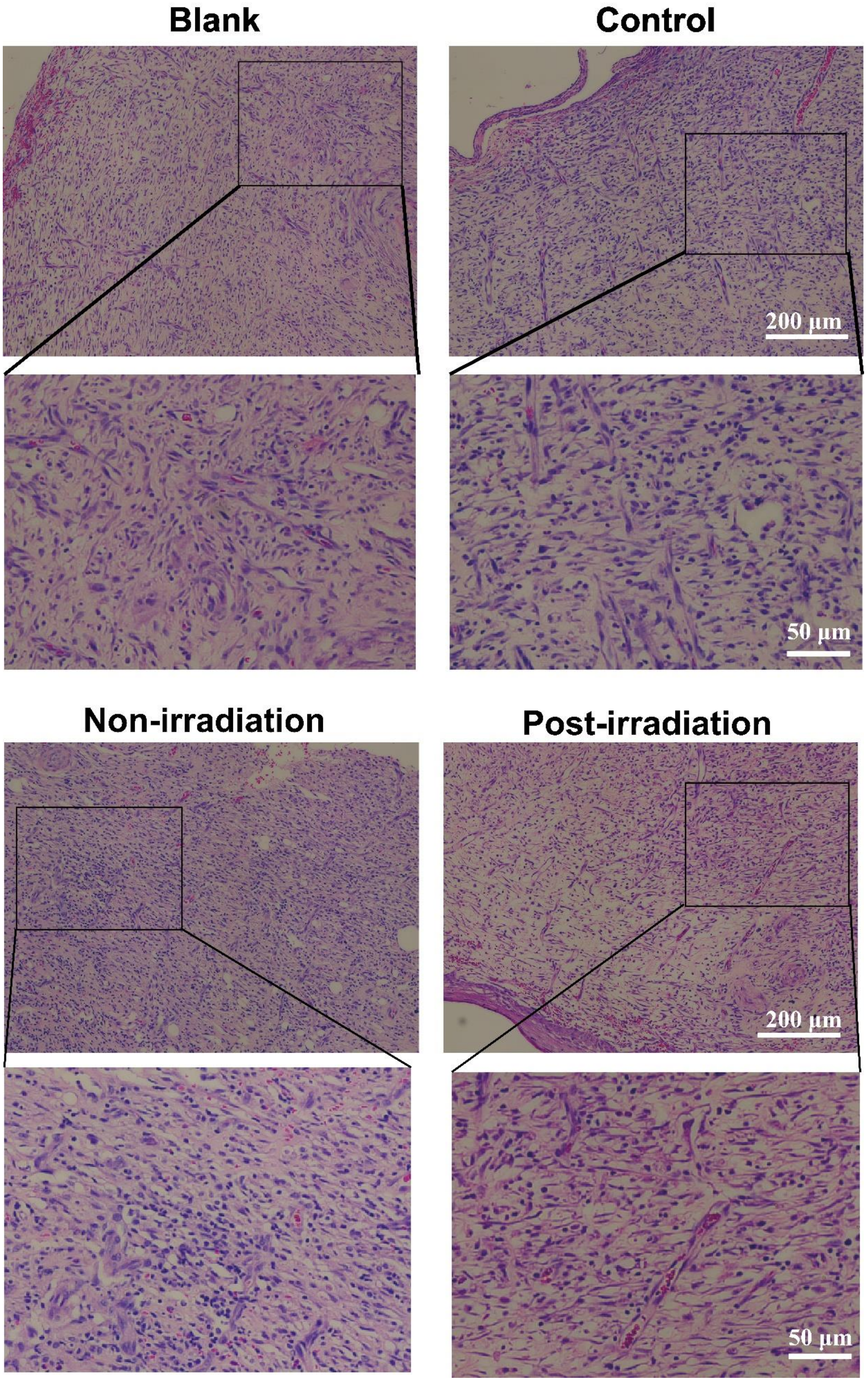

Figure S23 Body weight of differently treated rats as a function of time after initiating treatment. N = 3, data are shown as the mean ± standard deviation.

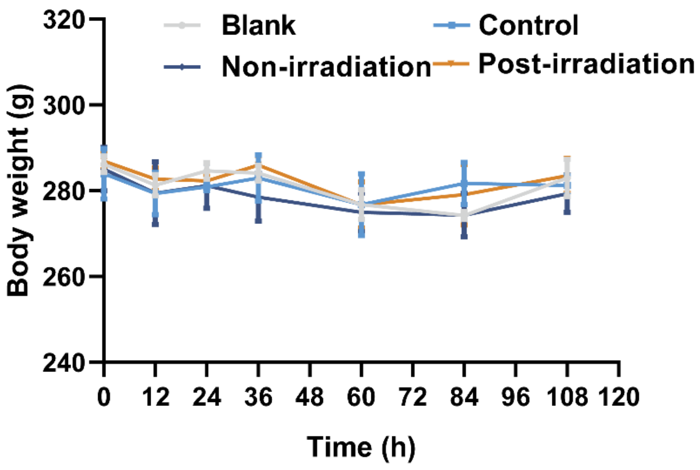

Supplement: Supplementary file 1 — Supporting Information [file ADVS-10-2206851-s001.pdf]
